# A Low-Frequency Oscillation Suppression Method for Regional Interconnected Power Systems with High-Permeability Wind Power

**DOI:** 10.3390/e26080689

**Published:** 2024-08-15

**Authors:** Yi Hu, Jinglin Luo, Kailin Yan, Tao Wang, Qingzhu Zeng, Tao Huang

**Affiliations:** 1 School of Electrical Engineering and Electronic Information, Xihua University, Chengdu 610039, China; 1220200001@xhu.edu.cn (Y.H.); 212022085800048@stu.xhu.edu.cn (K.Y.); wangatao2005@163.com (T.W.);; 2 Department of Energy, Politecnico di Torino, 10129 Turin, Italy

**Keywords:** interconnected power grid, damping characteristics, wind power permeability, parameter optimization, low-frequency oscillation suppression

## Abstract

With the integration of large-scale wind power into the power grid, the impact on system stability, especially the issue of low-frequency oscillations caused by small disturbances, is becoming increasingly prominent. Therefore, this paper proposes a damping quantitative analysis method for regional interconnected power systems incorporating large-scale wind power. Using the cross-entropy particle swarm optimization (CE-PSO) algorithm, the control parameters of wind turbines are optimized to suppress low-frequency oscillations in interconnected systems. The method begins with the state equation of the interconnected power system in two regions; it deduces the characteristic polynomial of the interconnected system, including wind farms, and takes into account the influence of wind power integration on the electrical connectivity of the system. Subsequently, the influence of wind turbine control parameters on the system is quantified, and a quantitative analysis model of the impact of wind power integration on system damping characteristics is constructed. Based on this, an optimization model for wind turbine control parameters is established, and the CE-PSO algorithm is utilized to achieve suppression of low-frequency oscillations in interconnected power grids with wind power integration. Finally, the accuracy and effectiveness of the proposed method are verified through a typical electromagnetic transient simulation model of the two-region interconnected power system.

## 1. Introduction

With the integration of large-scale wind power into the power grid, the internal structure of the power grid is becoming more and more complex. The new energy grid-connected equipment with power electronics as the interface significantly changes the operation characteristics of the power system dominated by the synchronous machine and reduces the transient stability of the power system, especially the small disturbance stability problem represented by low-frequency oscillation [1,2]. The large-scale grid connection of wind power makes the power oscillation characteristics of the system more complex, and the system damping changes significantly compared with the traditional power grid [3,4,5]. Therefore, it is necessary to deeply analyze the effect of wind power grid-connected characteristics on system damping characteristics, reveal the influence mechanism of wind power grid-connected scale and wind turbine control characteristics on system oscillation stability, and then select the corresponding low-frequency oscillation suppression method.

At present, many scholars have carried out related research on the small signal stability of the wind power grid based on the damping characteristics of the system. The influence of wind power grid connection on the low-frequency oscillation of the system is studied by using the transient simulation model outlined in References [4,6,7,8,9]. The doubly fed induction generator (DFIG) is converted into an equivalent traditional synchronous motor in Reference [6], and the small disturbance stability of the system with wind power permeability is analyzed under the condition of high and low wind speed. Reference [7] models wind turbines and analyzes the stability of the system based on the damping ratio from the perspectives of wind farm grid connection points, grid capacity, and transmission power of interconnection lines. In Reference [8], research on small disturbance stability of wind turbines connected to the grid is classified into two categories: one is to directly replace synchronous units and the other is to add a wind turbine dynamic model in system simulation, and the mechanisms of these two kinds of research results are compared and analyzed. Reference [9] used a 16-machine equivalent simulation system for feature analysis, which analyzed the impact of the depth of the penetration of wind generation on the low-frequency infrastructure modes of the interconnected power system. The mathematical model of dynamic interaction between a synchronous generator and wind turbine is established in Reference [4]. The influence of wind turbine supplementary active power control (SAPC) on low-frequency oscillation is analyzed, and the analysis conclusion is verified by nonlinear simulation of simplified two-region system and IEEE 39-bus system. Most of these research establishes small signal stability analysis models with wind power and then builds specific electromagnetic transient simulation models to analyze the impact of wind power grid connection on the system but cannot directly quantitatively analyze the influence mechanism of wind turbine grid connection on system damping characteristics.

Therefore, the whole mathematical model of the wind turbine incorporated into the power system is constructed in References [5,10,11,12,13,14] to analyze the oscillation characteristics. The dynamic energy model of a DFIG with PLL is derived in References [5,10]. From a dissipative energy perspective, the influence of the interaction between the DFIG and power grid on the low-frequency oscillation of the system is revealed. Reference [11] analyzes the influence of the virtual inertia of the DFIG participating in the frequency regulation of the system on the power grid dissipative energy perspective. The results show that the interaction between the virtual inertia of the wind turbine and the grid side may induce system oscillation and divergence. Reference [12] established a closed-loop dynamic model of a power system with DFIG grid connection and found that when the open-loop oscillation modes of the DFIG subsystem and the remaining subsystems have similar frequencies, it may cause mutual repulsion of the closed-loop system oscillation modes, leading to a significant reduction in system damping. The degree of its impact is positively correlated with wind power permeability. Reference [13] quantified the dynamic interaction introduced by wind turbines using damping torque analysis, finding that the dynamic interaction between new energy and power system is usually very weak and that the influence of this dynamic interaction on low-frequency oscillation damping is much smaller than that of power flow factors. Reference [14] performed single-machine infinite equivalence on interconnected systems and analyzed the impact of wind power inertia control on system damping by obtaining the characteristic root variation in the inertia time constant of the wind power integration into the system. Although the above studies have modeled the whole grid-connected system including wind power, most of them consider incorporating wind power into the infinite grid for analysis and do not consider the influence of wind turbines on the low-frequency oscillation of the inter-regional power grid within the system.

The above studies all indicate that with the integration of large-scale wind power, the power system will exhibit weak damping and low inertia characteristics, and the risk of low-frequency oscillation will increase. Therefore, some studies have further proposed corresponding optimization strategies for wind turbine control parameters based on the analysis of the impact mechanism of wind power integration on low-frequency oscillations, in order to improve the system damping characteristics resulting from wind turbine integration. For example, Reference [15] proposes a virtual synchronous generator (VSG) parameter optimization method based on a data-driven depth deterministic gradient strategy. With node voltage, branch current and other simulation data as input, VSG control parameters are adjusted online to achieve the target of low-frequency oscillation suppression in a model-free manner. By simplifying and reconstructing the full-order linearization model of a type 4 wind turbine, Reference [16] established a current source damping torque model suitable for analyzing the stability of a DC voltage loop and phase-locked loop, and realized the low-frequency oscillation suppression of a type 4 wind turbine by optimizing the phase and amplitude parameters of the damping transfer loop. References [17,18] analyzed the influence of a power oscillation damper (POD) on low-frequency oscillation of a power system containing a DFIG, and realized the low-frequency oscillation suppression of power system by constructing an optimization model of POD parameters. At present, this kind of research is mainly carried out for a specific or a single additional controller and cannot take into account the suppression effect of multiple control parameter adjustment in the wind turbine on the low-frequency oscillation between power grid regions.

In light of this, the present paper constructs a quantitative analysis model for system damping, specifically targeting interconnected power systems with large-scale wind power integration. Based on this model, we have achieved the suppression of low-frequency oscillations in interconnected systems through the optimization of wind turbine control parameters. The primary contributions of the proposed method encompass:

(1) Derivation of the state-space equations for the interconnected system incorporating wind power, mapping wind turbine control parameters into the characteristic polynomial, and thereby enabling the quantitative analysis of wind turbine control characteristics within the system damping analysis model;
(2) Utilization of the quantitative damping analysis model to intuitively reveal the mechanism by which wind power penetration influences low-frequency oscillations in interconnected power grids;
(3) Introduction of the CE-PSO (Cultural Evolutionary Particle Swarm Optimization) algorithm to optimize wind turbine control parameters, achieving optimal control of low-frequency oscillations.

The remainder of this paper is organized as follows. Section 2 derives the state-space equations for the interconnected system with wind power integration. Section 3 establishes the analysis model for damping characteristics in regionally interconnected power grids. Section 4 presents the damping control strategy based on the CE-PSO optimization algorithm. Section 5 provides simulation analysis of a case study. Finally, Section 6 concludes the paper.

## 2. State Equation of the Regionally Interconnected Power Grid with Wind Farms

**Figure 1:**
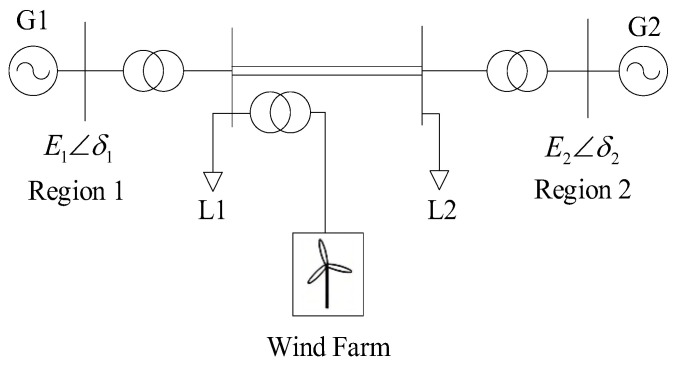
Wind power connected to two-machine interconnection system.

Figure 1 shows the simplified model of the two-region interconnection system. Assuming that the transmission direction from Region 1 to Region 2 is positive, the equivalent units of the two power grids adopt the second-order generator model; the generator’s transient reactance $X_{d}$, the transient electromotive force $E_{1}$ and the mechanical power $P_{m}$ are constant; and the load adopts the constant impedance model.

In the absence of wind power generation access, the state equation of the interconnected system can be obtained through the generator swing equation [19] as follows:

(1) $$\left\{\begin{array}{l} \overset{•}{\Delta \delta_{1}}=\Delta \omega_{1}\\ \overset{•}{\Delta \delta_{2}}=\Delta \omega_{2}\\ \overset{•}{\Delta \omega_{1}}=\frac{1}{M_{1}}(\Delta P_{1M}-\Delta P_{1e}-D_{1}\Delta \omega_{1})\\ \overset{•}{\Delta \omega_{2}}=\frac{1}{M_{2}}(\Delta P_{2M}-\Delta P_{2e}-D_{2}\Delta \omega_{2}) \end{array}\right.$$

where $\Delta \delta_{1}$ and $\Delta \delta_{2}$ represent the power angle increment of generators G1 and G2; $\Delta \omega_{1}$ and $\Delta \omega_{2}$ are speed increments; $\Delta P_{1M}$ and $\Delta P_{2M}$ are mechanical power increments; $\Delta P_{1e}$ and $\Delta P_{2e}$ are electromagnetic power increments; $M_{1}$ and $M_{2}$ are inertial time constants; $D_{1}$ and $D_{2}$ are damping torque coefficients.

According to the power flow calculation, the electromagnetic power expression of G1 and G2 output in Figure 1 without wind power can be obtained, and the synchronous torque coefficient of the generator is $K_{ij}=\partial P_{ie}/\partial \delta_{j}$. Assuming the mechanical power is constant, i.e., $\Delta P_{1M}=\Delta P_{2M}=0$, Equation (1) can be converted to:

(2) $$\left[\begin{array}{l} \overset{•}{\Delta \delta_{1}}\\ \overset{•}{\Delta \delta_{2}}\\ \overset{•}{\Delta \omega_{1}}\\ \overset{•}{\Delta \omega_{2}} \end{array}\right]=\left[\begin{array}{cccc} 0 & 0 & 1 & 0\\ 0 & 0 & 0 & 1\\ -\frac{K_{11}}{M_{1}} & -\frac{K_{12}}{M_{1}} & -\frac{D_{1}}{M_{1}} & 0\\ -\frac{K_{21}}{M_{2}} & -\frac{K_{22}}{M_{2}} & 0 & -\frac{D_{2}}{M_{2}} \end{array}\right]\cdot \left[\begin{array}{l} \Delta \delta_{1}\\ \Delta \delta_{2}\\ \Delta \omega_{1}\\ \Delta \omega_{2} \end{array}\right]=A_{S}\left[\begin{array}{l} \Delta \delta_{1}\\ \Delta \delta_{2}\\ \Delta \omega_{1}\\ \Delta \omega_{2} \end{array}\right]$$

The eigenvalue of the characteristic polynomial $\left|A_{s}-\lambda I\right|=0$ in the system state matrix can effectively reflect the low-frequency oscillation characteristics of the system, so the matrix change in Equation (2) can be obtained:

(3) $$\begin{array}{l} \lambda^{4}+(\frac{D_{1}}{M_{1}}+\frac{D_{2}}{M_{2}})\cdot \lambda^{3}+(\frac{D_{1}D_{2}}{M_{1}M_{2}}+\frac{K_{11}}{M_{1}}+\frac{K_{22}}{M_{2}})\cdot \lambda^{2}\\ +\frac{1}{M_{1}M_{2}}(D_{1}K_{22}+D_{2}K_{11})\cdot \lambda =0 \end{array}$$

**Figure 2:**
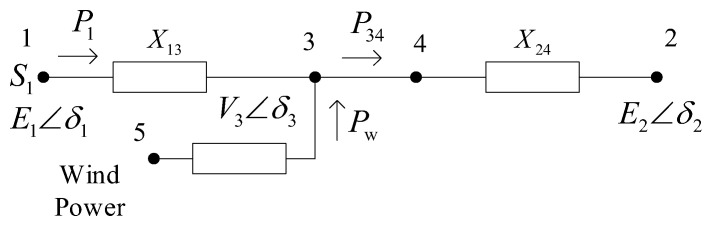
Wind power grid connected equivalent circuit.

The system shown in Figure 1 can then be simplified to the equivalent circuit shown in Figure 2.

Based on the above equation, considering only the active power $P_{1}$ injected into the node, we can derive:

(4) $$P_{1}=\frac{\left|{\vec{E}}_{1}\right|\cdot \left|{\vec{V}}_{3}\right|}{X_{13}}\cos(\delta_{1}-\delta_{3})=\frac{E_{1}V_{3}}{X_{13}}\cos(\delta_{1}-\delta_{3})$$

where $P_{1}$ represents the active power injected by node 1. The voltage phasor at node 1 is ${\vec{E}}_{1}=E_{1}∠\delta_{1}$, and the voltage phasor at node 3 is ${\vec{V}}_{3}=V_{3}∠\delta_{3}$.

Considering the incremental equation of the active power injected into the node for the above equation, we can obtain the following:

(5) $$\Delta P_{1}=\frac{E_{1}V_{3}}{X_{13}}\cos(\delta_{10}-\delta_{30})(\Delta \delta_{1}-\Delta \delta_{3})$$

where $P_{1}$ represents the active power injected by node 1. The voltage phasor at node 1 is ${\vec{E}}_{1}=E_{1}∠\delta_{1}$, and the voltage phasor at node 3 is ${\vec{V}}_{3}=V_{3}∠\delta_{3}$.

Considering the large-scale wind power grid connection, ignoring the line power loss and the line’s own resistance, the electromagnetic power increment equation in Regions 1 and 2 is as follows:

(6) $$\left\{\begin{array}{l} \Delta P_{1e}=\frac{E_{1}V_{30}}{X_{13}}\cos(\delta_{10}-\delta_{30})(\Delta \delta_{1}-\Delta \delta_{3})=k_{1}(\Delta \delta_{1}-\Delta \delta_{3})\\ \Delta P_{2e}=\frac{E_{2}V_{30}}{X_{23}}\cos(\delta_{30}-\delta_{20})(\Delta \delta_{3}-\Delta \delta_{2})=k_{2}(\Delta \delta_{3}-\Delta \delta_{2}) \end{array}\right.$$

where $V_{3}$ is the voltage increment of the wind power junction point; $\delta_{3}$ is the phase increment of the wind power junction point; $X_{13}$ and $X_{23}$ are the reactance of generators G1 and G2 to the junction point of wind power, respectively. The variable subscript 0 represents the initial value.

At this time, without considering the load changes and network consumption, the following can be obtained:

(7) $$\Delta P_{1e}+\Delta P_{w}=\Delta P_{2e}$$

where $\Delta P_{w}$ is the active power output of the wind farm.

In the interconnected power system, if the frequency of the wind farm junction point slightly changes to $\Delta \omega_{B}$ and the dynamic frequency coefficient of the wind farm is $g_{1}$, then the active power output of the wind farm is $\Delta P_{w}=g_{1}\Delta \omega_{B}$. In the interconnected power grid, the frequency variation in the junction point of the wind farm can be represented by the frequency variation in the generator close to it. When the wind farm is located in Region 1, $\Delta \omega_{B}=g_{2}\Delta \omega_{1}$; when the wind farm is located in Region 2, $\Delta \omega_{B}=g_{3}\Delta \omega_{1}$.

According to the small-value oscillation micro-interference theory, when the rotor has small-value oscillation, the frequency of the node is determined by the frequency of the nearby generator, i.e., $g_{2}\approx 1$ and $g_{3}\approx 1$. By combining Equations (6) and (7), the non-state variables in the middle are eliminated. Subsequently, when the wind farm is located in either the power feeding region or the power accepting region, the state equation of the interconnected system can be obtained by incorporating Equation (1).

(8) $$\left[\begin{array}{l} \overset{•}{\Delta \delta_{1}}\\ \overset{•}{\Delta \delta_{2}}\\ \overset{•}{\Delta \omega_{1}}\\ \overset{•}{\Delta \omega_{2}} \end{array}\right]=\left[\begin{array}{cccc} 0 & 0 & 1 & 0\\ 0 & 0 & 0 & 1\\ -\frac{k_{1}k_{2}}{M_{1}(k_{1}+k_{2})} & -\frac{k_{1}k_{2}}{M_{1}(k_{1}+k_{2})} & -\frac{D_{1}}{M_{1}}+\frac{k_{1}g_{1}}{M_{1}(k_{1}+k_{2})} & 0\\ -\frac{k_{1}k_{2}}{M_{2}(k_{1}+k_{2})} & -\frac{k_{1}k_{2}}{M_{2}(k_{1}+k_{2})} & \frac{k_{1}g_{1}}{M_{2}(k_{1}+k_{2})} & -\frac{D_{2}}{M_{2}} \end{array}\right]\cdot \left[\begin{array}{l} \Delta \delta_{1}\\ \Delta \delta_{2}\\ \Delta \omega_{1}\\ \Delta \omega_{2} \end{array}\right]$$

(9) $$\left[\begin{array}{l} \overset{•}{\Delta \delta_{1}}\\ \overset{•}{\Delta \delta_{2}}\\ \overset{•}{\Delta \omega_{1}}\\ \overset{•}{\Delta \omega_{2}} \end{array}\right]=\left[\begin{array}{cccc} 0 & 0 & 1 & 0\\ 0 & 0 & 0 & 1\\ -\frac{k_{1}k_{2}}{M_{1}(k_{1}+k_{2})} & -\frac{k_{1}k_{2}}{M_{1}(k_{1}+k_{2})} & -\frac{D_{1}}{M_{1}} & \frac{k_{2}g_{1}}{M_{1}(k_{1}+k_{2})}\\ -\frac{k_{1}k_{2}}{M_{2}(k_{1}+k_{2})} & -\frac{k_{1}k_{2}}{M_{2}(k_{1}+k_{2})} & 0 & -\frac{D_{2}}{M_{2}}+\frac{k_{2}g_{1}}{M_{2}(k_{1}+k_{2})} \end{array}\right]\cdot \left[\begin{array}{l} \Delta \delta_{1}\\ \Delta \delta_{2}\\ \Delta \omega_{1}\\ \Delta \omega_{2} \end{array}\right]$$

Taking the wind farm in the power feeding region as an example, the characteristic equation of Equation (8) is as follows:

(10) $$\begin{array}{l} \lambda^{4}+(\frac{D_{1}}{M_{1}}+\frac{D_{2}}{M_{2}}-\frac{k_{1}g_{1}}{M_{1}(k_{1}+k_{2})})\cdot \lambda^{3}+(\frac{D_{1}D_{2}}{M_{1}M_{2}}+\frac{k_{1}k_{2}}{M_{1}(k_{1}+k_{2})}-\frac{k_{1}k_{2}}{M_{2}(k_{1}+k_{2})}\\ -\frac{D_{2}k_{1}g_{1}}{M_{1}M_{2}(k_{1}+k_{2})})\cdot \lambda^{2}+\frac{k_{1}k_{2}}{M_{1}M_{2}(k_{1}+k_{2})}(D_{2}-D_{1})\cdot \lambda =0 \end{array}$$

As can be seen from Equation (10), when the junction point of the wind farm changes, $X_{13}$ and $X_{23}$ will change, resulting in changes in $k_{1}$ and $k_{2}$, thus changing the system damping. When the output power of the wind farm changes, $\Delta P_{w}$ will also change, which will cause a change in system damping. According to the same analysis, when the wind farm is in the accepting region, the output power of the wind farm and the junction point will also change the damping characteristics of the interconnected power grid.

To sum up, for interconnected power systems with large-scale wind power grid connection, the damping characteristics of the system are closely related to multiple aspects such as wind farm junction points, wind farm dynamic frequency characteristic coefficients, and wind farm output power (i.e., wind power permeability).

## 3. Analysis of Damping Characteristics of Regional Interconnected Power Grid

### 3.1. Influence of Wind Power Permeability

From the electromagnetic power increment equation in Regions 1 and 2, the expressions of $k_{1}$ and $k_{2}$ are as follows:

(11) $$\left\{\begin{array}{l} k_{1}=\frac{E_{1}V_{30}}{X_{13}}\cos(\delta_{10}-\delta_{30})=\frac{E_{1}V_{30}}{X_{13}}\cos\delta_{130}\\ k_{2}=-\frac{E_{2}V_{30}}{X_{23}}\cos(\delta_{30}-\delta_{20})=-\frac{E_{2}V_{30}}{X_{23}}\cos\delta_{320} \end{array}\right.$$

Assuming that the transmission power of the connecting line is $P_{34}$, the wind farm has a constant power factor, and there is no reactive power exchange with the grid; then, based on power balance, it can be concluded that:

(12) $$P_{1}+P_{w}=P_{2}$$

The permeability of wind power is defined as η%:

(13) $$\eta \%=\frac{P_{w}}{P_{w}+P_{1}+P_{2}}$$

Since wind power is accessed in Region 1, regardless of its influence on the reactance between nodes 2 and 4, the output characteristics of wind power can be considered equivalent to variable reactance. Consequently, when only the active power output of the wind farm is taken into account, we can obtain:

(14) $$X_{35}=-\frac{V_{3}^{2}}{P_{w}}=-\frac{1}{2\eta \%P_{34}}$$

After the wind power is equivalent to variable reactance, the total reactance between nodes 1 and 3 can be obtained:

(15) $$X_{13\Sigma }=\frac{X_{13}X_{35}}{X_{13}+X_{35}}$$

Based on the equivalent circuit model shown in the figure above, for AC lines 1 to 3, by neglecting the line resistance and only considering the line reactance $X_{13}$, the longitudinal and transverse components of the node voltage drop can be calculated as follows:

(16) $$\left\{\begin{array}{l} \Delta V=R_{13}I\cos\varphi +X_{13}I\sin\varphi \\ \delta V=X_{13}I\cos\varphi -R_{13}I\sin\varphi \end{array}\right.$$

Taking the voltage phasor $V_{3}$ as the reference axis and neglecting the circuit resistance R, we obtain:

(17) $$\left\{\begin{array}{l} \Delta V=\frac{P_{1}R_{13}+Q_{1}X_{13}}{V_{3}}=\frac{Q_{1}X_{13}}{V_{3}}\\ \delta V=\frac{P_{1}X_{13}-Q_{1}R_{13}}{V_{3}}=\frac{P_{1}X_{13}}{V_{3}} \end{array}\right.$$

According to the definition of node voltage drop, we have:

(18) $$E_{1}=\sqrt{{\left(V_{3}+\Delta V\right)}^{2}+{\left(\delta V\right)}^{2}}$$

Then, based on the above equation, we can derive:

(19) $$E_{1}=\sqrt{{\left(V_{3}+\frac{Q_{1}X_{13}}{V_{3}}\right)}^{2}+{\left(\frac{P_{1}X_{13}}{V_{3}}\right)}^{2}}$$

Thus, the Equation (20) can be obtained through the node voltage drop equation:

(20) $$\left\{\begin{array}{l} E_{1}=\sqrt{{\left(V_{3}+\frac{Q_{1}X_{13}}{V_{3}}\right)}^{2}+{\left(\frac{P_{1}X_{13}}{V_{3}}\right)}^{2}}\\ =\sqrt{{\left(V_{3}+\frac{P_{34}\tan\varphi X_{13}}{V_{3}}\right)}^{2}+{\left(\frac{\left(1-2\eta \%\right)P_{34}X_{13}}{V_{3}}\right)}^{2}}\\ E_{2}=\sqrt{{\left(V_{3}-\frac{Q_{2}X_{24}}{V_{3}}\right)}^{2}+{\left(\frac{P_{2}X_{24}}{V_{3}}\right)}^{2}}\\ =\sqrt{{\left(V_{3}-\frac{P_{34}\tan\varphi X_{24}}{V_{3}}\right)}^{2}+{\left(\frac{P_{34}X_{24}}{V_{3}}\right)}^{2}} \end{array}\right.$$

The voltage vector phase difference is:

(21) $$\left\{\begin{array}{l} \delta_{130}=\arctan\frac{\frac{\left(1-2\eta \%\right)P_{34}X_{13}}{V_{3}}}{V_{3}+\frac{P_{34}\tan\varphi X_{13}}{V_{3}}}\\ \delta_{320}=\arctan\frac{\frac{P_{34}X_{24}}{V_{3}}}{V_{3}-\frac{P_{34}\tan\varphi X_{24}}{V_{3}}} \end{array}\right.$$

(22) $$\left\{\begin{array}{l} k_{1}=\frac{E_{1}V_{30}}{X_{13}}\cos(\delta_{10}-\delta_{30})=\frac{E_{1}V_{3}}{X_{13}}\cos\delta_{130}\\ k_{2}=-\frac{E_{2}V_{30}}{X_{23}}\cos(\delta_{30}-\delta_{20})=-\frac{E_{2}V_{3}}{X_{23}}\cos\delta_{320} \end{array}\right.$$

where δ is a function of wind power permeability, from which the relationship between wind power permeability and $k_{1}$ and $k_{2}$ can be obtained.

### 3.2. Dynamic Frequency Characteristic Coefficient of the Wind Turbine

**Figure 3:**
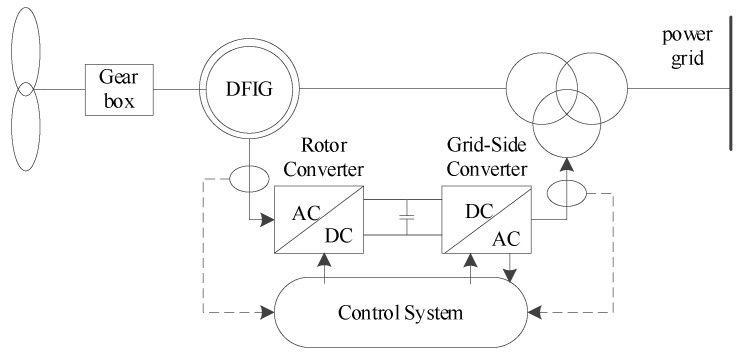
Structure diagram of DFIG.

The stator windings of DFIGs are directly connected to the power grid, while the rotor windings are connected to the grid through a converter. The frequency, voltage, amplitude, and phase of the rotor winding power supply are automatically adjusted by the converter according to operational requirements. This allows the generator set to achieve constant-frequency power generation at different rotational speeds. Figure 3 illustrates the grid integration structure of the DFIG.

The mathematical model of a DFIG in the abc coordinate system is converted to the d-q coordinate system by the Park transformation. Assuming voltage and current symmetry in the three-phase winding, the flux equation of the doubly fed induction motor in the synchronous rotating d-q coordinate system is as follows:

(23) $$\left\{\begin{array}{l} \psi_{\mathrm{sd}}=L_{s}i_{\mathrm{sd}}-L_{m}i_{\mathrm{rd}}\\ \psi_{\mathrm{sq}}=L_{s}i_{\mathrm{sq}}-L_{m}i_{\mathrm{rq}} \end{array}\right.$$

(24) $$\left\{\begin{array}{l} \psi_{\mathrm{rd}}=-L_{m}i_{\mathrm{sd}}+L_{r}i_{\mathrm{rd}}\\ \psi_{\mathrm{rq}}=-L_{m}i_{\mathrm{sq}}+L_{r}i_{\mathrm{rq}} \end{array}\right.$$

In the equation, $\psi_{\mathrm{sd}}$, $\psi_{\mathrm{sq}}$, $\psi_{\mathrm{rd}}$ and $\psi_{\mathrm{rd}}$ are the components of the stator flux d and q axis and the rotor flux d and q axis, respectively. $i_{\mathrm{sd}}$, $i_{\mathrm{sq}}$, $i_{\mathrm{rd}}$ and $i_{\mathrm{rq}}$ are the current components of stator d and q axes and rotor d and q axes, respectively. $L_{s}$, $L_{r}$ and $L_{m}$ are stator leakage inductance, rotor leakage inductance and mutual excitation inductance, respectively, in the d-q coordinate system.

In the synchronous rotating d-q coordinate system, the stator and rotor voltage equations of the DFIG are as follows:

(25) $$\left\{\begin{array}{l} u_{\mathrm{sd}}=-R_{s}i_{\mathrm{sd}}-p\psi_{\mathrm{sd}}-\omega_{1}\psi_{\mathrm{sq}}\\ u_{\mathrm{sq}}=-R_{s}i_{\mathrm{sq}}-p\psi_{\mathrm{sq}}+\omega_{1}\psi_{\mathrm{sq}} \end{array}\right.$$

(26) $$\left\{\begin{array}{l} u_{\mathrm{rd}}=R_{r}i_{\mathrm{rd}}+p\psi_{\mathrm{rd}}-(\omega_{1}-\omega_{r})\psi_{\mathrm{rq}}\\ u_{\mathrm{rq}}=R_{r}i_{\mathrm{rq}}+p\psi_{\mathrm{rq}}+(\omega_{1}-\omega_{r})\psi_{\mathrm{rd}} \end{array}\right.$$

In the equation: $R_{s}$ and $R_{r}$, respectively, are the stator and rotor winding resistance; $\omega_{1}$ and $\omega_{r}$ represent synchronous rotation angular speed and rotor rotation angular speed; and p is a differential operator.

When the direction of the stator flux vector is the same as the direction of the rotating coordinate system d axis, the stator resistance, $R_{s}=0$, can be obtained:

(27) $$\left\{\begin{array}{l} u_{\mathrm{sd}}=0\\ u_{\mathrm{sq}}=\left|U_{s}\right|=U_{s} \end{array}\right.$$

(28) $$\left\{\begin{array}{l} \psi_{\mathrm{sd}}=\psi_{s}=-\frac{U_{s}}{\omega_{1}}\\ \psi_{\mathrm{sq}}=0 \end{array}\right.$$

where $U_{s}$ represents the stator voltage.

Equations (23) and (28) can then be combined to substituted into (24). Letting $\sigma =L_{r}-L_{m}^{2}/L_{s}$ represent the rotor flux equation expressed by rotor current and stator voltage, it can then be substituted into (26) for further simplification.

(29) $$\left\{\begin{array}{l} u_{\mathrm{rd}}=R_{r}i_{\mathrm{rd}}+p\sigma i_{\mathrm{rd}}-(\omega_{1}-\omega_{r})(\sigma i_{\mathrm{rq}})\\ u_{\mathrm{rq}}=R_{r}i_{\mathrm{rq}}+p\sigma i_{\mathrm{rq}}+(\omega_{1}-\omega_{r})(-\frac{L_{m}}{\omega_{1}L_{s}}U_{s}+\sigma i_{\mathrm{rd}}) \end{array}\right.$$

The converter on the rotor side of the DFIG employs constant active power and constant AC voltage control. This includes dual-loop control, where the d-axis utilizes constant active power control with an outer power loop and an inner current loop, while the q-axis employs constant AC voltage control.

**Figure 4:**
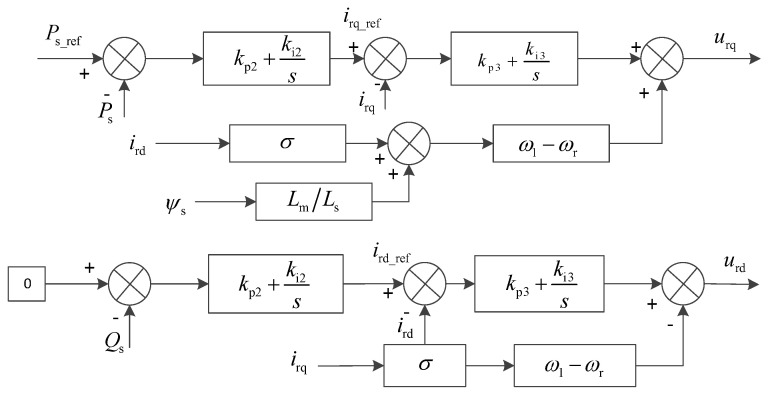
Control block diagram of DFIG rotor side converter.

Figure 4 shows the control block diagram of the DFIG rotor side converter, where $P_{s\_\mathrm{ref}}$ and $P_{s}$ are the reference and actual values of the stator active power, $i_{\mathrm{rd}\_\mathrm{ref}}$ and $i_{\mathrm{rq}\_\mathrm{ref}}$ are the reference values of the stator current d-axis and q-axis, $k_{p2}$ and $k_{d2}$ are the PD parameters of the power outer loop control of the rotor-side converter, and $k_{p3}$ and $k_{d3}$ are the PD parameters of the current inner loop control of the rotor-side converter.

From Figure 4, it can be concluded that:

(30) $$\left\{\begin{array}{l} u_{\mathrm{rd}}=\left(i_{\mathrm{rd}\_\mathrm{ref}}-i_{\mathrm{rd}}\right)\left(k_{p3}-\frac{k_{d3}}{s}\right)-(\omega_{1}-\omega_{r})i_{\mathrm{rq}}\sigma \\ u_{\mathrm{rq}}=\left(i_{\mathrm{rq}\_\mathrm{ref}}-i_{\mathrm{rq}}\right)\left(k_{p3}-\frac{k_{d3}}{s}\right)+(\omega_{1}-\omega_{r})\left(i_{\mathrm{rq}}\sigma +\frac{L_{m}}{\omega_{1}L_{s}}U_{s}\right) \end{array}\right.$$

By combining Equations (29) and (30), the transfer function of the current of d-axis and q-axis on the rotor side changing with the current reference value can be obtained as follows:

(31) $$G_{1}\left(s\right)=\frac{i_{\mathrm{rd}}}{i_{\mathrm{rd-ref}}}=\frac{i_{\mathrm{rq}}}{i_{\mathrm{rq-ref}}}=\frac{sk_{p3}+k_{d3}}{s^{2}\sigma +s\left(R_{r}+k_{p3}\right)+k_{d3}}$$

Based on the relationship between the increment of stator active power and the increment of rotor current in the DFIG, it can be concluded that:

(32) $$\Delta P_{s}=\Delta i_{\mathrm{sd}}u_{\mathrm{sd}}+\Delta i_{\mathrm{sq}}u_{\mathrm{sq}}=\frac{U_{s}L_{m}}{L_{s}}\Delta i_{\mathrm{rq}}$$

where $i_{\mathrm{sd}}$ and $i_{\mathrm{sq}}$ represent the current components of the stator on the d-axis and q-axis, respectively; and $u_{\mathrm{sd}}$ and $u_{\mathrm{sq}}$ represent the voltage components of the stator on the d-axis and q-axis, respectively.

According to Figure 4 as well as Equations (31) and (32), the transfer function of the actual change value and the reference change value of the stator active power can be obtained as follows:

(33) $$G_{p}\left(s\right)=\frac{\Delta P_{s}}{\Delta P_{\mathrm{s-ref}}}=\frac{\left(sk_{p2}+k_{d2}\right)G_{1}\left(S\right)U_{s}L_{m}}{sL_{s}+\left(sk_{p2}+k_{d2}\right)G_{1}\left(S\right)U_{s}L_{m}}$$

**Figure 5:**
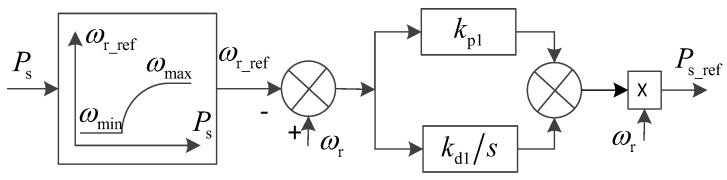
The speed control diagram of DFIG.

Figure 5 shows the speed control diagram of the DFIG, which controls the reference value of the stator active power of the wind turbine through the speed control of the wind turbine.

In Figure 5, $k_{p1}$ and $k_{d1}$ are PD parameters of speed control; $\omega_{\mathrm{rmin}}$ and $\omega_{\mathrm{rmax}}$ are the minimum and maximum operating speed, respectively; and $\omega_{\mathrm{r-ref}}$ is the reference value of the rotation angular speed of the rotor.

**Figure 6:**
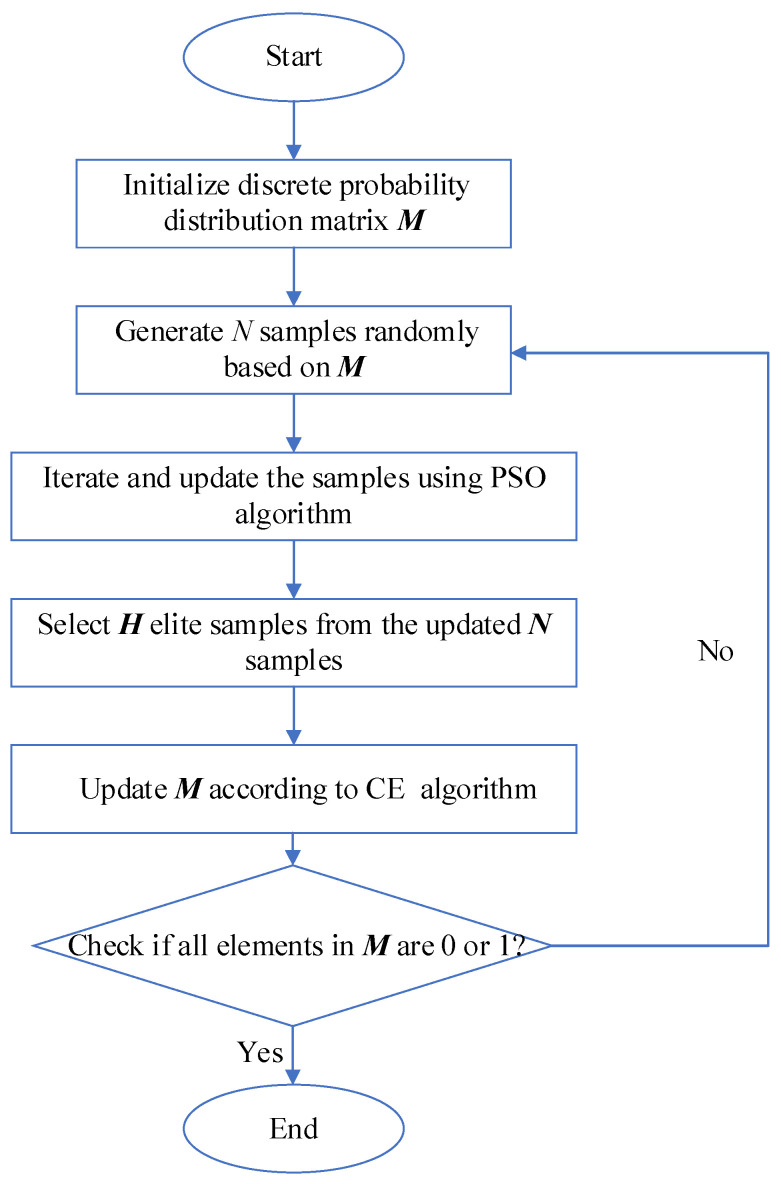
CE-PSO algorithm flow chart.

It can be obtained from Figure 6:

(34) $$P_{s-\mathrm{ref}}=\omega_{r}\left(\omega_{r}-\omega_{\mathrm{r-ref}}\right)\left(k_{p1}+\frac{k_{d1}}{s}\right)$$

Equations (33) and (34) are linearized to obtain:

(35) $$\left\{\begin{array}{l} \Delta \omega_{\mathrm{r-ref}}=-1.5P_{s0}\Delta P_{s}+1.59\Delta P_{s}\\ \Delta P_{\mathrm{s-ref}}=\left(2\omega_{r0}\Delta \omega_{r}-\omega_{\mathrm{r-ref}0}\Delta \omega_{r}-\omega_{r0}\Delta \omega_{r-\mathrm{ref}}\right)\left(k_{p1}+\frac{k_{d1}}{s}\right) \end{array}\right.$$

Sorting (35), the following can be obtained:

(36) $$\left\{\begin{array}{l} \Delta \omega_{\mathrm{r-ref}}=\left(-1.5P_{s0}+1.59\right)\Delta P_{s}\\ \Delta P_{\mathrm{s-ref}}=\left[\left(2\omega_{r0}-\omega_{\mathrm{r-ref}0}\right)\Delta \omega_{r}-\omega_{r0}\Delta \omega_{r-\mathrm{ref}}\right]\left(k_{p1}+\frac{k_{d1}}{s}\right) \end{array}\right.$$

By combining Equations (33) and (36), the transfer function of the change value of the stator active power of the wind turbine with the change value of the rotor speed can be obtained:

(37) $$G\left(s\right)=\frac{\Delta P_{s}}{\Delta \omega_{r}}=\frac{\left(2\omega_{r0}-\omega_{\mathrm{r-ref}0}\right)\left(sk_{p1}+k_{d1}\right)G_{p}\left(s\right)}{s+\omega_{r0}\left(-1.5P_{s0}+1.59\right)\left(sk_{p1}+k_{d1}\right)G_{p}\left(s\right)}$$

### 3.3. Low-Frequency Oscillation Characteristic Value of the System

Based on the modeling in Equation (10) and Section 3.2, the complete characteristic equation of the system after incorporating the wind turbine into the two regional interconnected power grids can be obtained as follows:

(38) $$\begin{array}{l} \lambda^{4}+(\frac{D_{1}}{M_{1}}+\frac{D_{2}}{M_{2}}-\frac{k_{1}G\left(\lambda \right)}{M_{1}(k_{1}+k_{2})})\lambda^{3}+(\frac{D_{1}D_{2}}{M_{1}M_{2}}+\frac{k_{1}k_{2}}{M_{1}(k_{1}+k_{2})}-\frac{k_{1}k_{2}}{M_{2}(k_{1}+k_{2})}-\frac{D_{2}k_{1}G\left(\lambda \right)}{M_{1}M_{2}(k_{1}+k_{2})})\cdot \lambda^{2}\\ +\frac{k_{1}k_{2}}{M_{1}M_{2}(k_{1}+k_{2})}(D_{2}-D_{1})\cdot \lambda =0 \end{array}$$

Obviously, the solution of this Equation (eigenvalue λ) can represent the low-frequency oscillation characteristics of the system. However, it can be seen that $G\left(\lambda \right)$ is a very complex polynomial, so it is very difficult to directly solve Equation (38), so the Lagrange function is adopted here for solving.

Assuming that the dominant low-frequency oscillation mode is λ=σ±jω, when the mode presents weak damping characteristics, it is considered that the eigenvalue is close to the virtual axis, i.e., σ≈0. At this time, λ=jω can be substituted into $G\left(\lambda \right)$ to obtain:

(39) $$\begin{array}{l} G(j\omega )\approx \frac{\Delta P_{s}}{\Delta \omega_{r}}=\frac{\left(2\omega_{r0}-\omega_{r0-\mathrm{ref}0})\cdot (sk_{p1}+k_{i1})\cdot G_{p}(j\omega \right)}{j\omega +\omega_{r0}(-1.7338P_{s0}+1.916)\cdot (sk_{p1}+k_{i1})\cdot G_{p}(j\omega )}\\ \approx M_{s1}+M_{d1}\lambda \end{array}$$

where $M_{s1}$ is the real part of G(jω) and $M_{d1}\lambda$ is the imaginary part of G(jω).

To make the analysis simple, it is assumed that the unit parameters and operating state of the wind farm are consistent, and the total output power of the wind farm is obtained by adding the output power of all the wind turbines; considering the wind power penetration is η%, let

(40) $$M_{s}+M_{d}\lambda =\eta \%\cdot P_{s}(M_{s1}+M_{d1}\lambda )$$

Bring Equation (40) into (38) to obtain

(41) $$\begin{array}{l} (1-\frac{k_{1}M_{d}}{M_{1}(k_{1}+k_{2})})\lambda^{3}+(\frac{D_{1}}{M_{1}}+\frac{D_{2}}{M_{2}}-\frac{k_{1}M_{s}}{M_{1}(k_{1}+k_{2})}-\frac{D_{2}k_{1}M_{d}}{M_{1}M_{2}(k_{1}+k_{2})})\cdot \lambda^{2}+(\frac{D_{1}D_{2}}{M_{1}M_{2}}+\frac{k_{1}k_{2}}{M_{1}(k_{1}+k_{2})}\\ -\frac{k_{1}k_{2}}{M_{2}(k_{1}+k_{2})}-\frac{D_{2}k_{1}M_{s}}{M_{1}M_{2}(k_{1}+k_{2})})\cdot \lambda^{1}+\frac{k_{1}k_{2}}{M_{1}M_{2}(k_{1}+k_{2})}(D_{2}-D_{1})=0 \end{array}$$

That is:

(42) $$a\lambda^{3}+b\lambda^{2}+c\lambda +d=0$$

where $a=1-\frac{k_{1}M_{d}}{M_{1}(k_{1}+k_{2})}$; $b=\frac{D_{1}}{M_{1}}+\frac{D_{2}}{M_{2}}-\frac{k_{1}M_{s}}{M_{1}(k_{1}+k_{2})}-\frac{D_{2}k_{1}M_{d}}{M_{1}M_{2}(k_{1}+k_{2})}$; $c=\frac{D_{1}D_{2}}{M_{1}M_{2}}+\frac{k_{1}k_{2}}{M_{1}(k_{1}+k_{2})}-\frac{k_{1}k_{2}}{M_{2}(k_{1}+k_{2})}-\frac{D_{2}k_{1}M_{s}}{M_{1}M_{2}(k_{1}+k_{2})}$; $d=\frac{k_{1}k_{2}}{M_{1}M_{2}(k_{1}+k_{2})}(D_{2}-D_{1})$.

Let $\lambda =y-\frac{b}{3a}$, substituting it into Equation (42) yields:

(43) $$y^{3}+py+q=0$$

where $p=\frac{3ac-b^{2}}{3a^{2}}$; $q=\frac{27a^{2}d-9abc+2b^{3}}{27a^{3}}$.

When Equation (43) satisfies condition $\Delta ={\left(\frac{q}{2}\right)}^{2}+{\left(\frac{p}{3}\right)}^{2}>0$, the cubic equation has two complex roots, and the expressions of the real part and imaginary part of the characteristic roots can be obtained as follows:

(44) $$\left\{\begin{array}{l} \sigma =-\frac{1}{2}\left(\sqrt[{3}]{-\frac{q}{2}+\sqrt{{\left(\frac{q}{2}\right)}^{2}+{\left(\frac{p}{3}\right)}^{3}}}+\sqrt[{3}]{-\frac{q}{2}-\sqrt{{\left(\frac{q}{2}\right)}^{2}+{\left(\frac{p}{3}\right)}^{3}}}\right)-\frac{b}{3a}\\ \omega =\frac{\sqrt{3}}{2}\left|\sqrt[{3}]{-\frac{q}{2}+\sqrt{{\left(\frac{q}{2}\right)}^{2}+{\left(\frac{p}{3}\right)}^{3}}}-\sqrt[{3}]{-\frac{q}{2}-\sqrt{{\left(\frac{q}{2}\right)}^{2}+{\left(\frac{p}{3}\right)}^{3}}}\right| \end{array}\right.$$

Since *p* and *q* are functions related to $M_{d}$ and $M_{s}$, and $M_{d}$ and $M_{s}$ are functions of ω, the real and imaginary parts need to be solved first by ω pairs. According to the imaginary part, ω is estimated by using the mapping method. First, the second term of Equation (43) is rewritten, which is

(45) $$F(\omega )=\frac{\sqrt{3}}{2}\left|\sqrt[{3}]{-\frac{q}{2}+\sqrt{{\left(\frac{q}{2}\right)}^{2}+{\left(\frac{p}{3}\right)}^{3}}}-\sqrt[{3}]{-\frac{q}{2}-\sqrt{{\left(\frac{q}{2}\right)}^{2}+{\left(\frac{p}{3}\right)}^{3}}}\right|-\omega$$

Based on the intersection point between the characteristic curve of F(ω) and the 0-axis, the frequency of low-frequency oscillation can be calculated, and then the real part can be estimated based on ω. Finally, the variation in the theoretical total damping can be estimated.

## 4. Optimization Control Strategy for Wind Power System Parameters Based on CE-PSO Algorithm

According to the analysis in Section 3, it can be seen that several groups of wind turbine control parameters have an impact on system damping. In order to improve the stability of the interconnected power grid, this paper considers optimizing the multiple control parameters of the wind turbine by building an optimization model, so as to improve the system damping characteristics.

### 4.1. Optimized Damping Control Model

This paper proposes an optimization control objective function by setting the desired damping ratio of the system and selecting the minimum difference between the damping ratio of the oscillation mode and the desired damping ratio under typical operating conditions.

(46) $$\begin{array}{cc} \min & J=\sum_{i=1}^{n}\Delta \zeta_{i.\max} \end{array}$$

where $\Delta \zeta_{i.\max}=\zeta_{i}-\zeta_{i.\mathrm{ref}}$ represents the maximum deviation value of the desired damping ratio under the i-th operating mode; $\zeta_{i.\mathrm{ref}}$ is the expected damping ratio; and $\zeta_{i}$ is the actual damping ratio.

Theoretically, the solutions to Equation (10) (i.e., the eigenvalues λ) can reveal the low-frequency oscillation behavior of the system. However, solving Equation (10) directly is exceedingly difficult; thus, we resort to utilizing the Lagrangian function for the solution.

Assuming the dominant low-frequency oscillation mode to be λ=σ±jω, when this mode exhibits weak damping characteristics, the eigenvalue is considered to be proximate to the imaginary axis, signifying σ≈0. Under this condition, λ=jω, and substituting these values into Equation (31) yields $G\left(\lambda \right)$ [20].

(47) $$G(j\omega )\approx \frac{\Delta P_{s}}{\Delta \omega_{r}}=\frac{\left(2\omega_{r0}-\omega_{r0\mathrm{-ref}0})\cdot (sk_{p1}+k_{d1})\cdot G_{2}(j\omega \right)}{j\omega +\omega_{r0}(-1.5P_{s0}+1.59)\cdot (sk_{p1}+k_{d1})\cdot G_{2}(j\omega )}\approx M_{s1}+M_{d1}\lambda$$

In the equation, $M_{s1}$ represents the real part of G(jω), while $M_{d1}$ represents the imaginary part of G(jω).

To simplify the analysis, it is assumed that the parameters and operating conditions of the wind turbine units within the wind farm are uniform. The total output power of the wind farm is taken as the sum of the output powers of the individual units. Assuming a wind power penetration rate of η%, let

(48) $$g_{1}\left(\lambda \right)=M_{s}+M_{d}\lambda =\eta \%\cdot P_{S}(M_{s1}+M_{d1}\lambda )$$

By substituting Equations (47) and (48) into Equation (10) and rearranging the terms, we obtain:

(49) $$\begin{array}{l} (1-\frac{k_{1}M_{d}}{M_{1}(k_{1}+k_{2})})\lambda^{3}+(\frac{D_{1}}{M_{1}}+\frac{D_{2}}{M_{2}}-\frac{k_{1}M_{s}}{M_{1}(k_{1}+k_{2})}-\frac{k_{1}M_{d}}{M_{1}M_{2}(k_{1}+k_{2})})\cdot \lambda^{2}\\ +(\frac{D_{1}D_{2}}{M_{1}M_{2}}+\frac{k_{1}k_{2}}{M_{1}(k_{1}+k_{2})}-\frac{k_{1}k_{2}}{M_{2}(k_{1}+k_{2})}-\frac{k_{1}M_{s}}{M_{1}M_{2}(k_{1}+k_{2})}+\frac{k_{1}^{2}k_{2}M_{d}}{M_{1}M_{2}{\left(k_{1}+k_{2}\right)}^{2}})\cdot \lambda^{1}\\ +\frac{k_{1}k_{2}}{M_{1}M_{2}(k_{1}+k_{2})}(D_{2}-D_{1}-\frac{k_{1}M_{s}}{k_{1}+k_{2}})=0 \end{array}$$

The damping ratio is an indicator used to evaluate the dynamic performance of a system. When the damping ratio is larger, the number of oscillations to reach a steady state will decrease. In power systems, the damping ratio is typically required to be no less than 0.05 s^−1^, and in some specific systems, the minimum damping ratio requirement is not less than 0.15 s^−1^. Here, $\zeta_{i.ref}=0.1s^{-1}$ is taken as the expected damping ratio.

The constraint conditions for each control parameter of the wind turbine should meet the following:

(50) $$\left\{\begin{array}{l} k_{p1.\min}<k_{p1}<k_{p1.\max}\\ k_{d1.\min}<k_{d1}<k_{d1.\max}\\ k_{p2.\min}<k_{p2}<k_{p2.\max}\\ k_{d2.\min}<k_{d2}<k_{d2.\max}\\ k_{p3.\min}<k_{p3}<k_{p3.\max}\\ k_{d3.\min}<k_{d3}<k_{d3.\max} \end{array}\right.$$

where $k_{d1.\min}$, $k_{d1.\max}$, $k_{d1.\min}$ and $k_{d1.\min}$ are the minimum and maximum values of $k_{d1}$ and $k_{p1}$, respectively; $k_{d2.\min}$, $k_{d2.\max}$, $k_{p2.\min}$ and $k_{p2.\max}$ are the minimum and maximum values of $k_{d2}$ and $k_{p2}$, respectively; and $k_{d3.\min}$, $k_{d3.\max}$, $k_{p3.\min}$, and $k_{p3.\max}$ are the minimum and maximum values of $k_{d3}$ and $k_{p3}$. respectively.

### 4.2. CE-PSO Optimization Method

In order to improve the traditional particle swarm optimization (PSO) algorithm’s tendency to fall into local optima in solving the damping control optimization model, this paper combines the cross-entropy (CE) algorithm and PSO algorithm and uses the CE-PSO algorithm to achieve control parameter optimization. This algorithm combines the discrete probability estimation of the cross-entropy algorithm and the random update strategy of the particle swarm optimization algorithm, which can greatly improve the global optimization ability and optimization effect of complex models [21,22].

The essence of the CE algorithm is to transform an optimization problem into a probability estimation problem. Assuming J is a real-valued function defined on a finite state set X, the CE algorithm converts the optimization problem of finding the maximum value of this function into a probability estimation problem.

(51) $$k_{d1.\min}J(X^{*})=\gamma^{*}=\underset{X\in \chi }{\max}J(X)$$

(52) $$l(\gamma )=p_{\beta }(J(X)\geq \gamma )=E_{\beta }I_{\left\{J(X)\geq \gamma \right\}}$$

Equation (51) represents the original optimization problem, which is to find the maximum value $\gamma^{*}$ of the function J within the statistical sample set X, as well as the state $X^{*}$ that makes J achieve the value of $\gamma^{*}$. Equation (52) represents the transformed estimation problem, where γ is a value close to $\gamma^{*}$, and under the parameter β, J(X) achieves the maximum probability, which is the expected value corresponding to the indicator function $I_{\left\{J(X)\geq \gamma \right\}}$.

To solve this problem, an unbiased estimation of l(γ) needs to be made, ultimately transforming the original optimization problem into the following maximum optimization problem:

(53) $$\underset{\beta }{\max}\;E_{\beta }I_{\left\{J(X)\geq \gamma \right\}}\ln f(X,\beta )=\underset{\beta }{\max}\frac{1}{N}\sum_{i=1}^{N}I_{\left\{J(X)\geq \gamma \right\}}\ln f(X,\beta )$$

This paper employs the CE-PSO algorithm to solve the established optimization model. Firstly, the CE algorithm is used to construct a discrete probability distribution function, which randomly generates the initial particle swarm for the PSO algorithm. Then, through the random walk and iterative optimization of the PSO algorithm, excellent samples are generated to update the discrete probability distribution function. This process is repeated continuously. The overall flowchart of the algorithm is shown in Figure 6.

The implementation steps of the CE-PSO algorithm are as follows:

(1) Convert the optimization variable matrix $X=(x_{ij}{)}_{m\times n}$ into a discrete probability distribution matrix $M=(m_{ij}{)}_{m\times n}$ and obtain a probability distribution function $k_{d1.\max}f(X,M)=\prod_{i=1}^{m}\prod_{j=1}^{n}m_{ij}^{g(i,j)}$ (where $g(i,j)=\left\{\begin{array}{l} 1,\;\;\;\;if\;\;\;\;X(j)=i\\ 0,\;\;\;\;else \end{array}\right.$ is part of the context). This realizes the initialization of the discrete probability distribution matrix M.
(2) Based on M, randomly generate N samples of $X_{1},X_{2},\ldots ,X_{N}$ and use them as the initial particles for the PSO algorithm.
(3) According to the particle position update equation of the PSO algorithm, perform multiple rounds of iterative updates on the N particles.
(4) Calculate the evaluation function S for the updated N particles, sort the particles in descending order based on their S values, and select the top H=θN particles as excellent samples.
(5) Based on the update equation of the discrete probability distribution matrix M:

(54) $$m_{ij}=(\sum_{k=1}^{H}g_{k}(i,j))/H$$

Using the H excellent samples to update the discrete probability distribution matrix M;

(6) Repeat steps (1) to (5) until all elements in M become 0 or 1, or the set iteration count is reached. At this point, the most excellent sample can be considered as the optimized optimal control parameters for the wind turbine.

## 5. Case Simulation

In this paper, the PSCAD platform is used to build a two-region interconnected power grid with wind farms connected to the grid shown in Figure 1, in which the parameters of a single fan are shown in Table 1, the load adopts a constant impedance model, the rated power of the equivalent generator $SG_{1}$ and $SG_{2}$ in the two regions are set to 900 MW, the rated power rate of a single typhoon is 2 MW, and the wind speed is set 11 m/s. As the number of wind turbines in the wind farm depends on the wind power access capacity, the wind power permeability of the system is adjusted by adjusting the number of wind turbines and the equivalent generator capacity of $SG_{1}$.

The speed control parameters and virtual inertia control parameters are analogous, and their value ranges can be referenced from the value range of virtual inertia.

(1) Calculation of the value range for $k_{p1}$

After the introduction of virtual inertia control, wind turbines exhibit similar inertia response and frequency regulation characteristics as equivalent synchronous generators. The unit regulating power $K_{G}$ of a traditional synchronous motor can be expressed as:

(55) $$K_{G}=\Delta P_{G}/\Delta \omega_{s}$$

where $\Delta P_{G}$ represents the change in generator power, $\Delta \omega_{s}$ represents the change in grid frequency, and $K_{G}$ signifies the static characteristic of active power.

From Equation (55), we can derive the following:

(56) $$\Delta P_{G}=K_{G}\Delta \omega_{s}$$

Upon comparing this with the power-frequency characteristic equation of a synchronous generator, it becomes evident that the value range of $k_{p1}$ can be referenced from the unit regulating power $K_{G}$ of a synchronous generator. The unit regulating power ranges for steam turbines and hydroturbines are, respectively, 20~33.3 and 25~50. Consequently, a suitable range for $k_{p1}$ in this context is chosen as 20~50.

(2) Calculation of the value range for $k_{d1}$

Based on the principle of energy conservation, we can utilize the conservation of rotor kinetic energy between wind turbine generators and synchronous generator sets to convert the rotor speed of the wind turbine generator to synchronous speed. Additionally, we must consider the safety of the system frequency and the rotor operation of the wind turbine generator to arrive at the following equation:

(57) $$K_{d1}=2H\frac{\omega_{e}^{2}}{\omega_{m}^{2}}\frac{\omega_{r}^{2}-\omega_{r0}^{2}}{\omega_{s}^{2}-\omega_{s0}^{2}}$$

where H represents the inertia time constant of the wind turbine generator, and $\omega_{e}$ is the base value of the system’s synchronous frequency.

In summary, the value range for $k_{d1}$ is 5~10.

The ranges for the rotor-side controller parameters can be inferred from references [23,24]:

The value range for $k_{p2}$ is 5~20. The value range for $k_{d2}$ is 1~10. The value range for $k_{p3}$ is 5~20. The value range for $k_{d3}$ is 1~10.

(58) $$\left\{\begin{array}{l} 20<k_{p1}<50\\ 5<k_{d1}<10\\ 5<k_{p2}<20\\ 1<k_{d2}<10\\ 5<k_{p3}<20\\ 1<k_{d3}<10 \end{array}\right.$$

**Figure 7:**
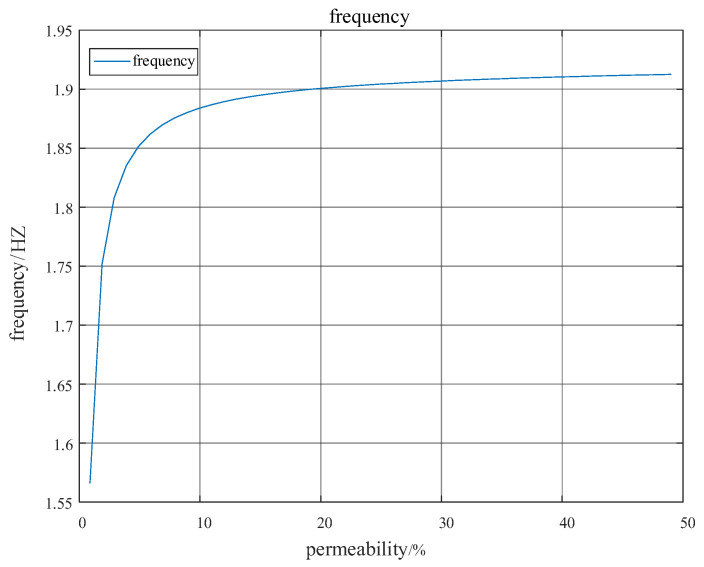
Variation in system oscillation frequency with wind power permeability.

- Quantitative analysis between low-frequency oscillation characteristics and wind power permeability of interconnected power grid;

According to the damping quantitative analysis method of the regional power grid with wind farm interconnection proposed in this paper, the quantitative relationship between low-frequency oscillation frequency and wind power permeability can be obtained as shown in Figure 7.

**Figure 8:**
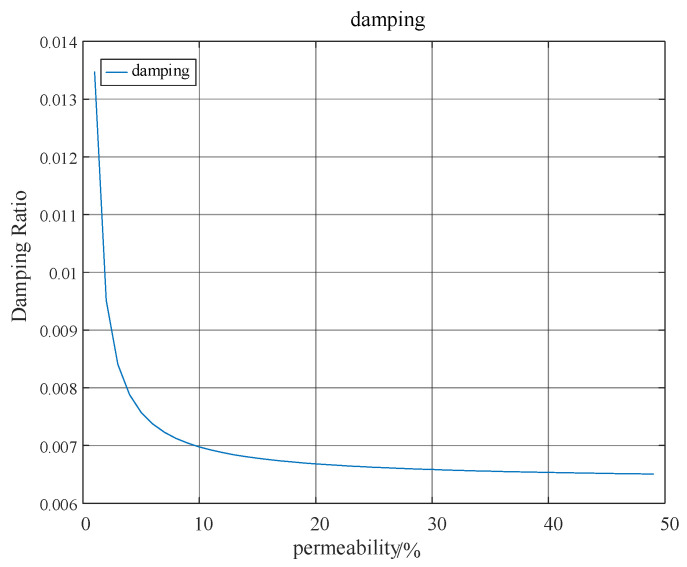
Variation in damping with wind power permeability.

At the same time, the quantitative relationship between the system oscillation damping ratio and the wind power permeability shown in Figure 8 can also be analyzed.

As evident from Figure 7 and Figure 8, the low-frequency oscillation between regional power grids exhibits an increasing trend with the augmentation of wind power penetration. Concurrently, the damping ratio declines as the level of penetration rises. Notably, when the penetration exceeds 30%, the diminishing effect on the damping ratio becomes less pronounced, and the system’s oscillation frequency essentially stabilizes.

- Quantitative analysis between low-frequency oscillation characteristics of interconnected power grid and control parameters of wind turbine;

By adjusting the number of wind turbines to set the wind power permeability of the interconnected power grid to 30%, according to the damping quantitative analysis method of the interconnected regional power grid with wind farms proposed in this paper, we can quantitatively analyze the relationship between different control parameters of wind turbines and inter-regional low-frequency oscillation characteristics.

**Figure 9:**
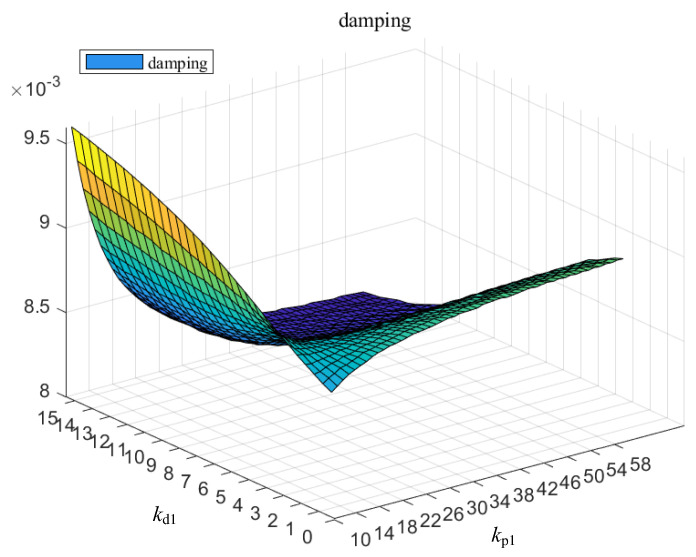
Variation in damping with speed control parameters.

Firstly, the relationship between the speed control PD parameters $k_{p1}$, $k_{d1}$ of the DFIG and the system oscillation damping ratio is shown in Figure 9 below.

It can be seen from Figure 9 that when $k_{p1}$ is less than 38, the damping increases with the increase in $k_{d1}$, and when $k_{p1}$ is greater than 38, the damping decreases with the increase in $k_{d1}$.

**Figure 10:**
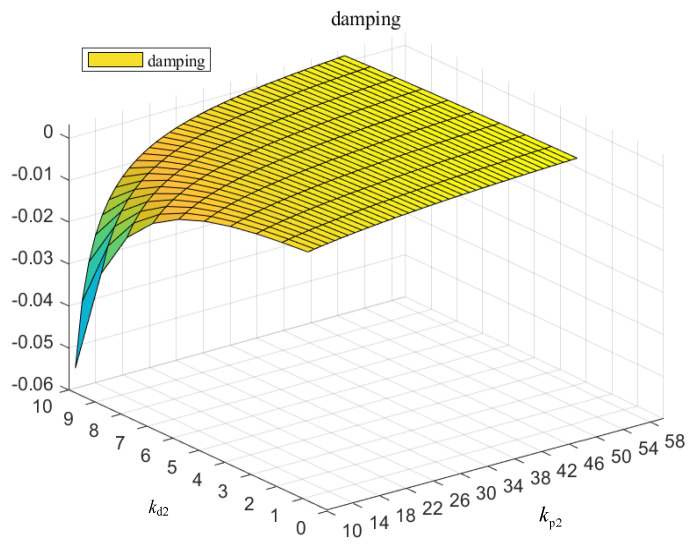
Variation in damping with control parameters of rotor power outer loop.

Secondly, the relationship between the power outer loop control PD parameters $k_{p2}$ and $k_{d2}$ of the DFIG rotor side converter and the system oscillation damping ratio is shown in Figure 10 below.

It can be seen from Figure 10 that improper PD parameters of the power outer loop control can easily lead to system damping cross-boundary instability, and the proportional parameters should be larger and the integral parameters should be smaller.

**Figure 11:**
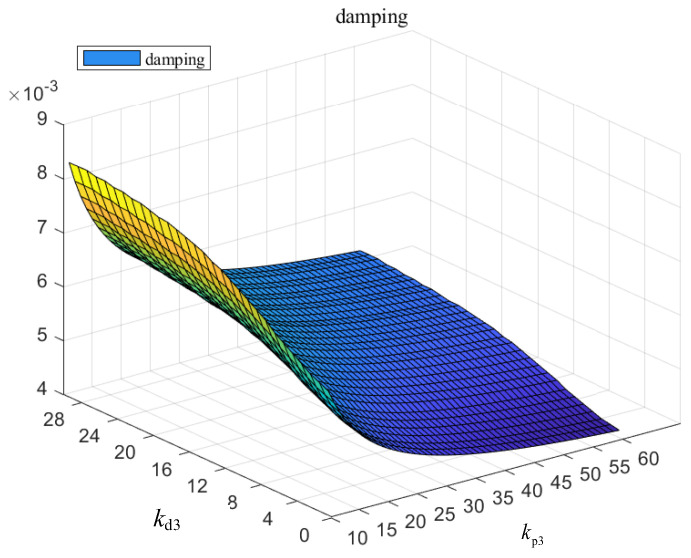
Variation in damping with control parameters of rotor current inner loop.

Furthermore, the relationship between the current inner loop control PD parameters $k_{p3}$ and $k_{d3}$ of the DFIG rotor side converter and the system oscillation damping ratio is shown in Figure 11 below.

It can be seen from Figure 11 that the damping decreases with the increase in the proportional parameters of the current inner loop control, and the damping increases with the increase in the integral parameters.

Through the analysis of the above cases, the interconnected power grid will produce different inter-regional oscillation modes under different wind power permeabilities. Different control parameters of wind turbines likewise exert varying influences on the system’s oscillation characteristics. Therefore, according to the damping ratio requirements and the specific wind power permeability, the reasonable control parameters of the wind turbine can be obtained by optimizing the model to improve the oscillation characteristics and realize the system damping control.

- Verification of damping control method;

Utilizing the CE-PSO algorithm, this paper optimizes the control parameters of the established model and ultimately obtains three optimized sets of PD control parameters, as shown in Table 2.

**Figure 12:**
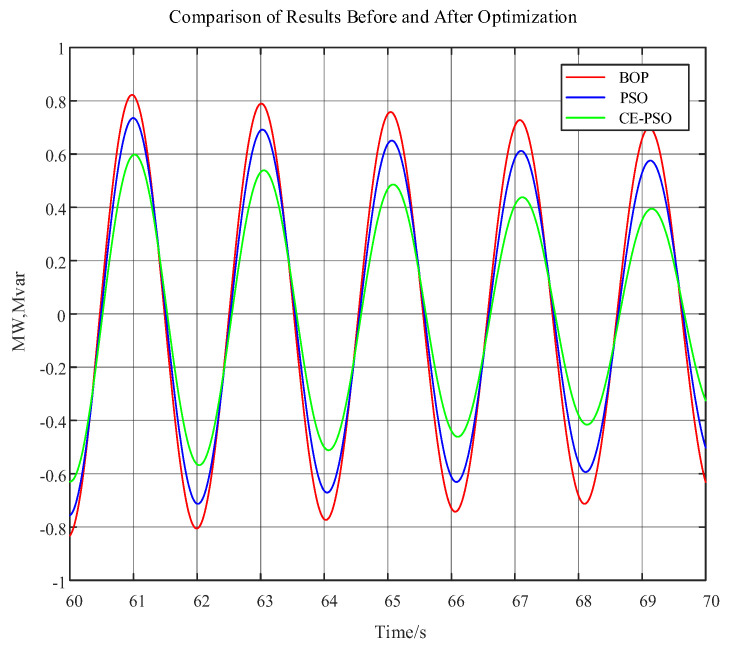
Active power oscillation curve of tie line 1 before and after optimization.

To verify that the low-frequency oscillations between power grid regions can be effectively suppressed after optimizing the aforementioned wind turbine control parameters, in this study, a comparative analysis of the low-frequency oscillation characteristics under before optimization conditions (BOP), after optimization using traditional PSO, and after optimization using CE-PSO was conducted. The results, which show the comparative oscillation characteristics, are presented in Figure 12 and Table 3 below, which summarize the key findings.

As clearly demonstrated in Figure 12 and Table 3, following the optimization of wind turbine control parameters, the oscillation decay between regions of the power grid is significantly accelerated compared to its pre-optimization state. Specifically, the system oscillation damping ratio is enhanced by a factor of 2.54 after the application of the proposed optimization method, indicating the effectiveness of the proposed wind turbine parameter optimization control approach in suppressing low-frequency oscillations across system regions. Furthermore, the optimization performance of the CE-PSO algorithm proposed in this paper is significantly superior to the traditional PSO algorithm. While both methods are able to ensure that the system damping ratio remains above 0.05 s^−1^ after optimizing the wind turbine control parameters, the system damping ratio achieved by the CE-PSO used in this study increased by 63.37% compared to the traditional PSO. This verifies the distinct advantages of the proposed method in this paper.

## 6. Conclusions

In this paper, a quantitative analysis method for damping in interconnected power grids with large-scale wind power integration is derived, and based on this, an inter-regional low-frequency oscillation suppression method for interconnected power grids is proposed, which is optimized based on wind turbine control parameters. Through theoretical and simulation analyses, the following conclusions are drawn:

1. Based on the quantitative analysis model, it is evident that as the wind power permeability increases, the impact on system damping gradually decreases. Moreover, there are significant differences in the influence of different wind turbine control parameters on system damping;
2. By constructing an optimization model to achieve coordinated optimization of all wind turbine control parameters, the low-frequency oscillation in the interconnected power grid can be effectively suppressed;
3. Regarding the optimization model for wind turbine control parameters, utilizing the CE-PSO algorithm can achieve even better optimization results, thus enhancing the effectiveness of suppressing low-frequency oscillations in the power grid.

The current study has not taken into account the impact of differences in control methods among different types of wind turbines on the low-frequency oscillations of the power system. In subsequent research, further investigations into this aspect should be conducted.

## Figures and Tables

**Table 1:** Wind turbine parameters.

Parameter	Value
Rated Power S/MVA	2
Rated Voltage $U_{s}$/kV	0.69
Stator Reactance $L_{s}$/pu	4.57
Stator Reactance $L_{r}$/pu	4.59
Field Reactance $L_{r}$/pu	4.5
Stator Resistance $R_{s}$/pu	0.0053
Rotor Resistance $R_{r}$/pu	0.00608
Mechanical Damping $D_{m}$/pu	0.01

**Table 2:** Optimization results of control parameters.

Control Parameters	Pre-Optimization Value	Post-Optimization Value
$k_{p1}$	13.53	36.42
$k_{d1}$	10.32	5.69
$k_{p2}$	2.33	7.24
$k_{d2}$	4.76	3.32
$k_{p3}$	5.78	17.61
$k_{d3}$	13.45	3.29

**Table 3:** Comparison of low-frequency oscillation characteristics under different methods.

Algorithms	Oscillation Frequency/Hz	Oscillation Attenuation Factor	Oscillation Amplitude	Initial Phase/Rad	Damping Ratio ξ/s^−1^
BOP	0.4918	−0.02	1.0241	−2.5114	0.0065
PSO	0.4918	−0.0301	1.0331	−2.5713	0.0101
CE-PSO	0.4918	−0.0501	1.0495	−2.6472	0.0165

## Data Availability

The data presented in this study are available on request from the corresponding author.
